# Psychology of Locke
*Locke's Essay on the Human Understanding. Abridged by J. Murray, LL.D. Dublin, 1852.


**Published:** 1854-07-01

**Authors:** 


### Art. III.—PSYCHOLOGY OF LOCKE.*

There is some danger, if any science real or pretended happens to become unusually popular, of other branches of knowledge being merged in it by its admirers and advocates. There was a time, not far distant, when this was exemplified by some of the phrenologists. The assertion was hazarded that we must henceforth seek the true psychology simply in the conformation of the cranium, not only as indicating each man's talents and character, but as also mapping out the human mind itself with its several powers. We trust this time is gone by; for the folly of seeking *out* of the mind what is *in it*, must be evident enough, surely, to every man who has carefully read any good work on the philosophy of the human faculties.

Our renowned countryman, John Locke, was an excellent example of freedom from all such pedantry. He had been intended for the medical profession; his predilection for it never ceased; and Dr.

* Locke's Essay on the Human Understanding. Abridged by J. Murray, LL.D. Dublin, 1852.

Sydenham, the most celebrated London physician of his day, did honour to Locke's judgment in allowing himself to be guided by some of his views, and dedicated to him his greatest work. Yet we look in vain, throughout the speculations of Locke, for any predominant tincture of his earlier pursuits: he seems on the contrary fully to have appreciated the independent character of those tracts of thought to which he afterwards so successfully devoted himself; and this is more evident in none than in that which related to the "Human Understanding." He allowed no physiological speculations to divert him from his inquiry into what our own mental experience teaches us; and Dugald Stewart has remarked that the "Essay" does not contain "a single passage savouring of the anatomical theatre or of the chemical laboratory."

The style of Locke is plain and unambitious. For a metaphysical work the language of the "Essay" may well be pronounced popular, as being marked by a matter-of-fact and straightforward spirit, and containing occasional passages of great though unadorned eloquence. Indeed, his figures have sometimes obscured his meaning, while his use of terms is not always uniform. The admirable precision and accuracy of Dugald Stewart, to whom scarcely any metaphysical writer whom we are acquainted with can be compared in this respect, was not to be expected in that age, and especially on such a subject. It required years and volumes of controversy, adjusting of misrepresentations, clearing up of difficulties, removal of ambiguities, collisions of systems, and clearances of old-established modes of expression borrowed from Aristotle, Descartes, and other writers, before such a style could be arrived at, in metaphysics, as that which honoured the English language in the works of the most learned British psychologist of his day.

There is a common prejudice against "Abridgments:" yet how many works on the subject of the Intellectual Philosophy would bear well, and with advantage to the reader, to be presented to him in an abbreviated form. The truth is that Abridgments are often vapid and useless from failing to convey a full and faithful picture of their original. This, for instance, is sure to be the case, almost, with all the attempts to exhibit in brief the philosophy of the Germans, in English. Yet what student would not have felt very much obliged to Kant, if instead of contenting himself with giving to the world his *Prolegomena*, containing certain heads of his great work the *Kritik der reinen Vernunft*, which after all require explanation by reference to the latter book itself, he had set himself to make a re-composition and digest of the *Kritik*, incorporating under their respective subjects the contents of the various appendices (*Anhänge*) which are so formidable and tiresome to the student, native or foreign, and saying once and for all what is often so tediously repeated, in various forms, and which would have been so capable of abridgment, especially by the hand of the celebrated writer himself? Though Locke's "Essay" is not fully open to a parallel criticism, we nevertheless think that where a clear apprehension of his system and doctrines is the sole object, a judicious abridgment might be far from useless. Such was attempted by John Wynne, of Oxford, in 1695, in the lifetime of Locke, to whom it was dedicated. It appears to be on the whole very well executed, the only fault which it suggests to us being that it omits certain parts of the work altogether; among which are the First Book, on "Innate Ideas," and the discussion on the "Will," in the Second; the abridger thinking the former involved in other parts, and the latter too long for his pages. This Abridgment, however, was only intended as an Introduction to the work itself, and Locke, as we learn from the preface, consented to its publication.

Dr. Murray proposes to "reduce the repetitions, diffusiveness, and irrelevant matter," which may be found in the "Essay," "not indeed to the lowest possible amount, but for the business of the Examination-hall" of Trinity College, Dublin, and the "advantage of the general reader;" and he defends the idea of such a compendium, by Locke's own candid remark, in his "Epistle to the Reader," that his work might be reduced to narrower compass, "the way it has been writ in, by catches, and many long intervals of interruption, being apt to cause some repetitions." The compiler also says that he has "sought to disembarrass the author's meaning in prolix or complicated passages, and generally to assist the connexion, by including within parenthesis-marks, and indicating by other definitive signs, those clauses and terms which seemed to require such specification." This "Abridgment," which is intended rather for the class-room than the library, seems to us to have been faithfully executed according to the compiler's plan; we have a quarrel with it only on the ground before-named, that it omits certain parts—which are indicated—the rule being not to admit anything excluded from the course prescribed to the students of Trinity College. Thus we miss, among other matter, the whole doctrine concerning "Innate Ideas." This omission we regard as a drawback, so far as it goes, from the usefulness of the work: for no mere private object or design, can easily reconcile us to the idea of not presenting a full and faithful picture of the corresponding original work, in an Abridgment, however much it may be a miniature.

The general estimate of Locke, in the "Preface" of the work before us, cannot be charged with indiscriminating partiality on the one hand, nor on the other with that unscrupulous and we may say unjust criticism with which our great metaphysician has been visited by not a few of his successors of various schools. For not only has his

"Sensualism," as it has improperly been called by the Germans,* generally, been condemned by them, as a whole, and treated almost as unceremoniously by the French Eclectic School; but it has met with little favour from some recent English writers, under the almost equally inappropriate name of "Sensationalism," which it would have been fair enough to apply to the Ideology of Condillac, and to the subsequent gross materialism of Cabanis and others; but which can never be fastened on Locke's system, except by those who take a most perverse and one-sided view of its plainly avowed and developed elements. In the preface of the present Abridgment, "originality, impartiality, candour, penetrating research, independent simplicity, and unpretending piety," are fairly claimed for the renowned author. "More dazzling theories than his have fascinated the eyes of men, more laboured researches have eluded them; but a more truth-loving inquirer after the knowledge both of God and of man than Locke, or a more trustworthy guide for youth toward, and (to a great extent) along *the proper road*, never yet professed either to search or to teach." In this view his work is a good medium of instruction to metaphysical study, as safe a practical text-book, as the youth of any community can enjoy." Yet, while thus favourably disposed towards Locke, and holding that the "Essay contains the germs of most of the metaphysical truths that have since received the most general assent," the compiler admits that it is "deficient in extent," and, in certain points of detail, "erroneous and even self-inconsistent." This brief estimate agrees very much with our own views.

It cannot be denied that Locke was the first who explicitly made an inquiry into the powers and phenomena of the inner man the *prime* object of metaphysical philosophy. Unlike Aristotle, he discarded all speculations which do not obviously come within the range of the human faculties: all ontological inquiries he expressly repudiated. Bacon's object was external nature, though he threw an incidental light on the method of all scientific pursuits, which was strong enough to lead the way to a psychology based on experience. Descartes, who was

* Even so recent, and in the main impartial a writer as Chalybäus, is not exempt from taking a similar sweeping view of Locke's psychology. He draws from it consequences scarcely less doleful than those which may be gathered from some of the eloquent lamentations of M. Cousin. He does not indeed intimate that it had any implicit relation to the Revolution of 1789, and the "Reign of Terror;" but he plainly charges it with leading to universal scepticism: Diess der Empirismus oder Sensualismus in seinen Grundzügen. . . . . Wir könnten mit jener Lehre nicht einmal Ordnung und Zusammenhang in der wirklichen Welt mit Sicherheit voraussetzen, geschweige uns mit Zuversicht zu dem Uebersinnlichen, zu den Ideen von Gott, Freiheit, Unsterblichkeit, erheben; da diese Ideen gar nicht auf sinnlichen Eindrücken beruhen, mithin nur als eine Fiction des dichtenden Verstandes, ohne alle äussere Berechtigung erscheinen würden.—*Historische Entwickelung*, u. s. w. Vorlesung I.

endowed with such rare talent for speculation, did not attempt a *system* of the phenomena of consciousness: he limited himself to certain questions, though these were no doubt fundamental. The same may be said of Spinoza, Malebranche, and even Leibnitz himself; who, much as he did to unfold certain great principles of human knowledge, owes his fame to his discovery, coevally with Newton, of the Differential Calculus. Locke, as is admitted by his able and severe critic, M. Cousin, "made the Origin of Ideas the grand problem of philosophy." His aim was the *Understanding*, so that the term "Intellectual Philosophy" is strictly applicable to his speculations. He made his inquiries into sensation, will, desire, and all other phenomena, subservient to the end of searching into the "Origin of our Ideas;" and he traced them all to two sources, "Sensation and Reflection."

Locke directs his First Book to the question of "Innate Notions:" the Second treats of Ideas, simple and complex; and under this head he also considers various Faculties and Operations of the mind: the Third Book is on Words: and the Fourth on Knowledge and Opinion. His brief statement of his general design is: "To inquire into the Original, Certainty, and Extent of Human Knowledge, together with the grounds and degrees of Belief, Opinion, and Assent." To a reader who may chance to have studied any of the more celebrated among the modern psychologists of the Scottish School, who have given a digested outline of the elements of the human mind, Locke's Table of Contents will appear heterogeneous and unsatisfactory, wanting in logical division, and defective in rigid analysis: but it should always be remembered that his object was not exactly to give a detailed and exhaustive system of all the phenomena of our consciousness, but to trace the manner in which our knowledge is acquired. This may account, in some measure, for the desultory air of many of the discussions, when viewed in relation to the natural desire which every reader has to refer everything to the landmarks of system: but it will still be found that the main object of ascertaining the sources of our knowledge, is rarely if ever lost sight of. Moreover, the freedom from the trammels of technical system which characterizes the work, may perhaps have been favourable to the independent way in which our great author enters afresh upon each successive topic of discussion, and to the natural method in which he analyses so many of the complications of thought.

In what sense did Locke use the term "Ideas," the origin of which is the subject of his great work? He himself replies: "whatsoever is the object of the understanding when a man thinks—whatever the mind can be employed about in thinking." Many of our readers will be aware that the term "Ideas" was originally used to signify the eternal patterns, or forms, supposed by Plato to exist in nature, and according to which God made all things. It was employed by Descartes, in a general signification, to stand for the objects of sense, imagination, and memory, as well as of the intellectual faculty itself. Locke evidently adopts the term in its widest meaning, for all the objects of human consciousness. So extensive is his sense of it, that we may say it is almost a synonyme for the phenomena of the mind in general. There is no doubt that Sir W. Hamilton's remarks (in his Paper on "Idealism" in the Edinburgh Review) respecting the fluctuations and vacillations of Locke's language, are substantially correct. He often fails to assign what in the Kantian metaphysic would be termed the "place" of the mental operations—that is the peculiar faculty to which they, in any given case, belong. He does not distinguish by his terms what he really intends to be distinct; but confounds object with operation, sensuous images of fancy with the notions proper to understanding, consciousness with perception and idea, and idea with notion, representation, and sense. The only way to deal with this laxity in the use of terms, which sometimes extends almost to materialism, if not quite — provided we literally interpret certain isolated passages and expressions—is to throw aside the rigid analysis of terms which has (advantageously indeed to psychological pursuits) occasioned so much controversy in the Scottish School, especially respecting idea and perception, and to understand Locke as often using these and other terms in a much more popular and general sense than they have since his time acquired in metaphysical writings. With Locke, the "Original of our Ideas," is in fact the same thing as the origin of our knowledge.

Now it is Locke's doctrine that all our ideas—all our knowledge—can come but from two sources, separately or combined, namely Sensation and Reflection. Whatever ambiguity there may be in his use of these terms, especially the latter, and notwithstanding the severe criticism which has visited this theory, we cannot but maintain that there is an obvious and important sense in which it is true that all our knowledge finds its way, by means of "experience," (as Locke also holds,) through these two channels. If any one were to ask what Locke means by "Reflection," we would reply, in general terms, he mainly means the power which enables us to *take notice of what passes in our minds;* and by "experience" he means the *aggregate of the particular instances* in which we are the subjects of sensation and reflection. Even Kant, the actual founder, if not the originator of the German ideal school—the great advocate for knowledge *à priori*, plainly lays down the position, as clearly as Locke himself, that all our knowledge begins with experience, and that the cognitive faculty itself is first called into action by the senses.* That it is also by means of the senses that we become acquainted with the properties and phenomena of the external world, no one who admits its existence can for a moment doubt, whatever dispute there may be about the precise mode in which we gain our irresistible conviction of its reality. And as to the knowledge of ourselves as conscious beings, the knowledge of our thoughts and inward processes, generally, it is equally evident that in order to gain this, we must turn the mental eye, as it were, within—in other words, observe what passes in our minds. The knowledge of the existence of God, our author derives from our knowledge of ourselves, or he thinks at least that it may be thus satisfactorily obtained. This then is what Locke means by Reflection, which, (so understood,) combined with Sensation, assuredly gives us all that we can know, from our natural powers, of the Universe, the Ego, and the Deity—the three great "Ideas" of the Transcendental School, and which it pronounces to comprise the whole sphere at which speculative reason aims.

But it has been asked, how can a certain class of ideas, those, namely, which appear to rise spontaneously in our minds, on certain occasions adapted to elicit them, as also those truths or impressions, intuitions, primary beliefs, first principles, axioms, self-evident truths, logical, or psychological (as, "I exist")—how can such truths as those which in the modern idealism would be termed "synthetic judgments *à priori*," be derived from Reflection, or from Reflection combined with Sensation? Is it thus that arises the idea of substance, for example, which I cannot but have, spontaneously, wherever I have experience of property—the idea of space as a necessary condition of all external objects—the idea of cause as the necessary antecedent of all change? These ideas have their corresponding *judgments*, which are synthetical, as containing more in the predicate than the mere analysis of the subject; for example, *every change must have a cause;* and there are many others, such as the true mathematical axioms, and in short all those propositions which are self-evident, and neither require nor admit proof—those which we term intuitive, in the English, not the German sense of intuition (*Anschauung*). Now, we are ready to admit that such knowledge as this is not, in one sense, gained from experience, either by means of the external or the internal faculty (Sensation or Reflection), or both combined; but we are just as clear that in another sense, and that the sense of Locke, such knowledge is thus gained.

* Dass alle unsere Erkenntniss mit der Erfahrung anfange, daran ist gar kein Zweifel; denn wodurch sollte das Erkenntnissvermögen sonst zur Ausübung erweckt werden, geschähe es nicht durch Gegenstände, die unsere Sinne rühren? u. s. w. —Kritik d. r. Vernunft, s. 695. Leipzig, 1838.

It is, in the strictest sense of the word, by "Experience," that zoologists have arrived at the conviction that "all horned animals have cloven feet;" yet no man feels any absurdity in supposing that the very same experience might possibly bring to light some remarkable exception to the rule: but in the case of the principle of causation, which tacitly underlies the assertions and even actions of very young children, we can conceive of no possibility of any such exception. We do not gain our knowledge of the general principle of causation as we gain a knowledge of the properties of the surrounding universe, namely by a gradual process. Has any event occurred—has any change of phenomena presented itself?—There *must* have been a cause. Convictions, principles, (call them what we may,) such as these, no doubt depend on our mental constitution itself; they are elicited as it were full-grown by their appropriate antecedents, at a period of life far too early for us to detect their rise, and they remain in the mind fixed and immovable throughout life. But, on the other hand, how do we arrive at the knowledge that such principles are necessary laws and conditions of thought? Surely by "Experience" and by nothing else—experience of our own consciousness. On this ground we are prepared to justify Dugald Stewart's expression—the "Inductive Philosophy of the Human Mind:" for he did not mean by this that our primary beliefs, or intuitive convictions were arrived at by a gradual process of induction: he only meant that we must reach a true system of the philosophy of man's mind, by carefully observing the facts of man's consciousness, and that we can then only say that a certain principle is a law of our own mind, and of men's minds, in general, when we find this principle operating uniformly and universally.

It should be remembered that notwithstanding Locke's awkward method of dealing with the undefined phantom of "Innate Ideas," he tacitly admits, over and over again, all that Descartes and Leibnitz really contended for, in connexion with this ill-chosen phrase—and this too, we may add, on Leibnitz's own showing, in his criticism of the "Essay." It is absurd to talk, as some of Locke's more modern critics have done, as though he meant to reject intuitive truth, either speculative or practical. The Essay contains striking illustrations of both. What he did mean was that when the external faculty has been brought into play by its appropriate objects, the internal faculty is also excited to action, and we are put into possession of a variety of truths which we should never otherwise have been able to know; or, in other words, Experience gives us, in Sensation and Reflection, all our knowledge. This is Locke's fundamental principle; and, in the sense in which he understood it, we doubt not that he is right. We may here add, that when Locke speaks of the mind as like white paper, or a *tabula rasa,* he can only, with fairness, be understood to mean what has just been said—that without the operation of the external and the internal faculties, the soul or mind would have no actual knowledge: he never intended to say that we have no natural tendencies, sensitive, intellectual, and moral, any more than he would have denied that the appearance of the writing on a fair sheet will depend in part on the texture of the paper. And even in attributing, with his frequent laxity of language, so large a portion of our "Ideas" to Sensation, as being "derived by the senses to the understanding," he is not wrong in claiming that priority for Sensation, in the natural order of the mind's procedure, which Kant himself clearly implies, when he asks, "by what means can our cognitive faculty be awakened to exercise, otherwise than by the objects which affect the senses?" *

Dugald Stewart, certainly one of the most competent of Locke's critics, admits that notwithstanding some unguarded expressions his fundamental doctrine "contains little that is reprehensible," as it may be interpreted to mean nothing more than that "the first *occasions* on which our various faculties are exercised, and the elements of all our knowledge acquired, may be traced ultimately to our intercourse with sensible objects;" and that these occasions are furnished, "either by impressions made on our external senses, or by the phenomena of sensation and thought of which we are conscious." † Yet the same distinguished writer, who was as candid as he was competent, adds that Locke's comments on the cardinal principle of his system, in different parts of the Essay, convey much more than is implied in the above interpretation; so that its author not only affirms that Sensation and Reflection furnish the occasions, in our experience, on which various elements of thought present themselves; but that these two faculties actually give to the mind all its simple ideas, in the "literal sense of the expression:" insomuch that they are all either immediate subjects of our consciousness, (such as the ideas we have of our own mental operations,) or copies of some quality perceived by external sense, another form of consciousness. We admit that no one can read the "Essay" attentively, and fail to observe that many such passages do occur. This has also been remarked by Dr. Reid, in his "Inquiry;" who regards Locke's fundamental position as being, "in plain English, that mankind neither do, nor can think of any thing but of the operations of their own minds."

Now it must unquestionably be granted that many of our ideas (to instance only those of space, time, substance, cause,) are not ideas of

* See note p. 346.

† Philosophical Essay, 1810, p. 18.

any operations of our minds whatever; yet we can think of these objects. But, as we have already intimated, the most strenuous defender of our great English philosopher, so far as he is defensible, —and he is so to a very wide and important extent—would not, or ought not at least, to contend for his accuracy in detail. Locke must be estimated by taking into view the time in which he lived—the comparative novelty of his subject as then unfamiliar to English literature, the scholasticism and the crude theories which had prevailed, the infancy of psychological and even of philological criticism which characterized the period in which he received his education. We must judge him by his broad views, strong sense, amazing superiority to reigning and inveterate prejudice—by the general outline and effect of the picture he has given us of the mind of man: in harmony of colouring, and accuracy of finish, he was undoubtedly defective. We cannot wholly defend him, by any means, from the home-thrusts of Reid, the dispassionate, candid, and gentlemanly criticism of Stewart; nor from the microscopic overhauling and the sharp and even cruel dissecting knife of M. Cousin, who has sometimes failed to do him the justice he deserved, partly perhaps from too much love of system; which has led the eclectic chief to identify Locke with a school the speculations of which he would have been the first to recoil from: for the really materialistic philosophers have not hesitated to propound the most unsupported and gratuitous general theories; but who more cautious than Locke?—who knew so well how to prefer doubt and even ignorance to uncertain or pretended knowledge?

That Locke formed an inaccurate estimate of a theory which, in his time, reigned extensively in the philosophical world, the theory of what were by a misnomer termed "Innate Ideas"—that he was not sufficiently informed as to what were really the Cartesian notions on this subject, especially as held by his contemporary Leibnitz, we will not deny: but the "Essay" was the most original, profound, and useful work on its subject, which modern times had ever yet had bequeathed to them. "It was not to be expected," says the late Professor Young, of Belfast, "that mental science should, like Minerva from the head of Jupiter, spring at once perfect from the lucubrations of one man." And we apprehend that much as controversy may do towards pointing out untenable theories, and adding greater precision to details, the difficulty which there is in arriving at an unexceptionable nomenclature —in short the ambiguity of *terms* (which, we fear, is next to incurable) will long continue to prove a formidable obstacle to the achievement of an Eclecticism which shall be perfectly satisfactory and unassailable. Sure we are that, at all events, the *spirit* in which Locke set about the task of propounding a philosophy of man's understanding—his caution in avoiding the danger of being misled into fine-spun theories by imagination under the guise of reason—his judicious estimate of the limitation of the human faculties—are invaluable lessons to all future inquirers in the same path. Indeed it has often occurred to us that we hardly appreciate the extent to which we are indebted to the writings of Locke on the various subjects which he handled, for the *practical sagacity* which even foreigners are ready to admit characterizes the intelligence of the English, as a nation. We certainly should be sorry to exchange the Baconian spirit and tendency of the "Essay," as a whole, for all the genius which, since the time of Kant, has been lavished, in Germany, on the attempt to seize upon the "absolute" and "unconditioned;" which has always ended like that of the boy who ran to catch the fragment of a rainbow while it was melting away.

We have little space for Locke's details. Indeed to do full justice to him, in regard to his views on the "Human Understanding," it would be necessary to refer, in connexion with the "Essay," at least to his controversy with Stillingfleet, and his examination of Malebranche. It is unfortunate that philosophers should so often misunderstand each other, in consequence of unnecessary laxity of expression on the one hand, or want of patient attention on the other, or both; as though the topics they treat of were not, in themselves, often difficult enough, without being further perplexed by misapprehension of each other's meaning. Descartes had spoken of certain ideas as *innatæ*, and *nées avec moi*. Voltaire, upon this, says, in his "Letters on the English Nation:" "Descartes asserted that the soul, at its coming into the body, is informed with the whole series of metaphysical notions, knowing God, and infinite space, and possessing all abstract ideas." But Descartes repeatedly explained that he "never meant that such ideas had an actual existence, or were distinct from the faculty of thinking."* Elsewhere, he states that all he means by having an innate idea, is "that we have within us a faculty for eliciting it."† Locke evidently misunderstood the true Cartesian theory of innate ideas; and, therefore, in his First Bock, he combated what was very much an error of language, which he had magnified into a monstrous and absurd hypothesis. This is evident from Leibnitz's candid admission that Locke's views were, in the final analysis, not incapable of being adjusted to the Cartesian doctrine. Unfortunately Locke died just at the time when Leibnitz was about to publish his *Nouveaux Essais;* in which he closely follows the topics of Locke's Essay, in a dialogue between

* Epist. 99. I.

† Denique cum dicimus ideam ubiquam esse innatam, intelligimus tantum nos habere in nobis facultatem illam eliciendi.—*Responsiones ad Objectiones.*

Philalèthe and Theophile. Had Locke survived, so as to have published a new edition, it is not unlikely that he might, in some important respects, have squared accounts with Leibnitz.* No doubt some of the Cartesians had used unguarded language with respect to the *κοιναὶ ἔννοιαι*; but passages occur in Locke, on this very subject, which might have been penned by either Descartes or Leibnitz.† Leibnitz himself represented Locke as maintaining the well-known scholastic principle: *Nihil est in intellectu quod non fuerit prius in sensu.* Yes, Locke did this in one way, but only in the way in which, as we have seen, it was substantially held even by Kant; and when Leibnitz added *nisi ipse intellectus*, he only expressed what Locke maintained in his doctrine of Reflection; though we do not altogether confess to the admiration with which the new edition of the aphorism has been spoken of in some quarters. If Locke is to be made, as he often is, an offender for a word, it is fair to say of his critic Leibnitz, that he mended the old school-saying rather clumsily; and that, in its new form, it is, as Mr. Lewes has remarked, very much like a man's saying "I have no money in my purse, except my purse itself"—an expression which, we dare say, might often be practically enough understood, though not quite precise for metaphysics. At all events, the amendment had no just bearing on the doctrine of Locke, however it might have hit the sensationalism of Gassendi and Hobbes, or the subsequent ideologism of Condillac. On the vexed subject of "Innate Ideas," Leibnitz himself, after considering various statements of Locke, ultimately declares, with much more candour than many writers, German, French, or even English of later date:—"I am inclined to believe that, fundamentally, Locke's views on this point are not different from my own."

We have already spoken of M. Cousin as one of the severest critics of Locke; and he is, at the same time, one of the most able. We are convinced, however, that even the materialistic passages and expressions which are too frequently to be found in the "Essay," particularly those which relate to Perception, by no means warrant Cousin in asserting that Locke is to be held responsible for the sensational doctrine of many who succeeded him. Indeed Cousin himself was quite aware that this school omitted one out of the two grand elements of Locke's theory; namely, the doctrine of "Reflection." Yet it must be granted, as appears to us, that no small ingenuity would sometimes be required for the task of defending Locke in a satisfactory manner from Cousin's animadversions. Compromises, palliations, and appeals to

* That this is probable may be gathered from Locke's candid avowal of his openness to conviction. Vid. Book I. chap. ii. § 28.

† *E.g.*, Essay, Book IV. chap. ii. § 13; and chap. xiii. § 3.

to the general tone and aim of the "Essay," are often necessary, to a considerable extent, if the object be to blunt the edge of criticism.

In consequence of the limitation of our space, we have been obliged to content ourselves chiefly with noticing Locke's most fundamental views. Among such are certainly the questions which we have now touched on—Is any knowledge "innate," or rather *intuitive?* Locke decidedly admitted the latter, but he did not make enough of it as a formal element in his system, though it was the real point in dispute between the Cartesians and himself. What are the *sources* of knowledge? Sensation and Reflection; and the latter separates Locke, by a wide interval, from the materialists; and even from Condillac, who, though he reduced all our mental phenomena to sensation, immediate or transformed, was himself an immaterialist. A third inquiry, scarcely less fundamental than those which relate to the general process by which our knowledge is acquired, and its sources, is that which aims to discover what is the analysis of our Knowledge. Locke defines knowledge to be "the perception of the agreement or disagreement of two ideas." In this alone, he says, it consists: "where this perception is, *there* is knowledge; and, where it is not, though we may fancy, guess, or believe, yet we always come short of knowledge." M. Cousin has minutely and rigidly criticized Locke's whole theory of knowledge: indeed, we think that, all things considered, (especially the unsettled state of philosophical terminology in his time—the lax and defective use of terms which was then common, and in which Locke participated,) Cousin's strictures on this whole subject are not free from a degree of hypercriticism, an extreme into which it is easy for a writer to fall who has so much real talent for microscopic dissection, and who moreover seems most at home in a style somewhat diffuse for the nature of the subject. Yet we cannot but admit, with Cousin, that Locke's definition of knowledge is inapplicable to many things which we are always said to *know*. The judgment, "I exist," cannot be brought into the same formula as other propositions—such as those which are arithmetical and geometrical, for example. For when I say *ego sum*, I assume my existence in the subject; and the predicate does not add to my conviction. We must of course admit, with Descartes, (who has beautifully discussed this point in his Meditations,) that our knowledge of our own existence is to us the most immediately certain of all things, and is intuitive, neither requiring nor admitting proof; for the *cogito ergo sum* of this distinguished philosopher was never intended by him to be a syllogism, but only a statement of the unassailable fact of consciousness—that it is in the process of thinking that our sense of self is forced on us.* It may perhaps be doubted after all whether Locke, could he have been interrogated, would not have excepted the intuitive and primary sense of our own existence, which is so identified with thought itself, from what he termed in a more popular and usual signification, *knowledge*, the principle of which, in general, he now proposed to give. Locke seems especially to have had in view our knowledge, as *acquired* by us. Our knowledge of our existence is only so far acquired as it is coeval with the first exercises of thought itself.

With regard to *logical* judgments, we conceive that Locke's definition, in the sense he meant, is substantially correct: for in every logical proposition there is a certain comparison of ideas, each idea being represented by a term; and as these ideas may be regarded as existing in the mind before we unite them in predication, the logical judgment may be said to be formed by the combination of ideas.† Cousin's objections to Locke's theory of knowledge, at least to the analysis which makes it consist in the "agreement or disagreement of ideas," apply only to judgments which we may designate as *psychological* in distinction from those which are logical. Thought itself may be viewed as a *sort of judging*. This is exemplified in all the spontaneous cognitions of the mind, when the actual presentations of perception and imagination produce a realization of the presence of their objects, without any process that can be called logical. In this way, the primitive psychological truth so well exhibited by Descartes, *je pense donc je suis*, is a psychological judgment; and it is no doubt one of those to which Locke's definition will not apply; for self is so presented in our acts of consciousness, from the time at least when the principle of thought has attained a distinct manifestation in the human being, that to know what we mean by *self*, is only to have that sense, impression, feeling, notion (call it what we may) of our own existence, which seems to blend itself with our whole consciousness, from a period too early for us to

* Descartes was charged with a *petitio principii* by Gassendi and others; but, as Spinoza his able commentator has remarked, he only meant to say that our thinking is attended with the conviction of our own existence. Descartes himself has said: "I think, therefore I am, or I exist, is not concluded by force of a syllogism, but as a thing self-evident." *Respons. ad Secund. Object.* Dr. Reid speaks of *Cogito ergo sum* as though designed to be a "logical argument," and seems rather reluctant to let Descartes off guiltless of the enthymeme.

† There is a sense in which even *Cogito ergo sum* might be regarded as a *logical argument,* though Descartes did not propound it as such. It might be thus regarded in opposition to that form of the German Idealism (the absolute form) which reduces everything to a *process of thought.* The strict Hegelian would say "I think," though he renounces all *real* existence. Should any one wish to express the necessity of real existence as the substratum of thought, and not merely that thought is a process synthesized under the term *ego,* he might of course use the enthymeme. But this was not the scope of Locke.

speculate on any genesis which it might be imagined to have. In this case, we may safely say that, psychologically, the subject and predicate are inseparable, if it be at all proper to use this language in reference to such a judgment. We must certainly agree with Cousin, that we did not arrive at the fundamental truth—" I exist," by first perceiving an "agreement" between the idea "*ego*," and the idea "*existence;*" but in a much more summary and immediate way; and, as we have intimated above, we hardly think that Locke himself, however unguardedly he may have expressed his general theory, would have deliberately included under it the judgment in question. We may add that he speaks on the subject of "our knowledge of our own existence," which he pronounces to be intuitive, very much indeed in the strain of Descartes himself.*

The limits of our space have obliged us chiefly to confine ourselves to a few main points, and we can now add little more. Of all the theories of Locke, perhaps that of "Personal Identity" has been regarded as the most singular. It was first criticised, as Sir W. Hamilton informs us, by John Sergeant, a name unknown to the historians of philosophy, till searched out by one of the most learned, laborious, and accurate of them all. Leibnitz, Bishop Butler, Reid, Stewart, Brown, and finally Cousin, have all animadverted on the "strange paradox" that a man is only the "same person so far as his consciousness (memory) reaches backwards." Now we are not about to defend Locke for having left us this whole remarkable discussion in the way in which it occurs in the "Essay :" but we may own ourselves to be somewhat surprised that so very little is said by his critics, in general, with respect to the use which he *professedly* made of the term *person.* By "person," he distinctly states that he did not understand the same material body, or even the same immaterial substance (soul,) nor both combined; he distinctly says that he does not mean by the "same person," the "same man;" so that Reid's elaborate argument, in which he proves (irrefragably enough, no doubt, provided the current sense of the term "person" be adopted,) that if any one had robbed an orchard when a boy, and remembered this act when he became a soldier, but forgot it after he was made a general, though when a general he remembered having become a soldier, he both would be and would not be the same "person" as a general that he was when a boy. Of course Locke would have said—All very true, if by "same person" you mean same man: but nothing can be clearer than that Locke understood "person" in a totally different sense. He lays down for himself the use of it as a "forensic term." In fact the whole discussion relates

* Essay: Book IV. chap. ix.

chiefly to the inquiry—when is a man to be held responsible for his actions, *in foro conscientiæ, in foro humano, in foro divino?* Cousin, in his Lectures on Locke's Philosophy, remarks as follows: "Locke has very clearly seen that where there is neither memory nor consciousness, there can be, for us, no idea of our personal identity; so that the sign, the character, the measure of personality, is consciousness. I cannot render too much praise to this part of Locke's theory: it attains and puts into light the true sign, the true character, the true measure of personality."* . . . . Now we apprehend that this was, in substance, what Locke really meant, infelicitously and paradoxically as he may have expressed it. Cousin adds that Locke has "confounded the condition of an idea, with this idea itself." Cousin, however, has not gone, with his usual minuteness, into the criticism of this part of Locke's "Essay;" nor has he specially noticed the "forensic" sense which Locke himself assigned to the term "person." We do not wish to be supposed to have any great fancy for Locke's mode of treating this subject; but we imagine that he meant to express, chiefly, that (in popular phrase) we must have been "*ourselves,*" at the time we did any action, in order to have it brought home to us; and that we can only realize, for ourselves, our own past agency, *as our own*, in proportion as we remember it. To enter into any inquiry as to the moral or the legal† bearings of Locke's doctrine, one way or the other, is what we cannot now attempt: we only mean to say that, as so much allowance is due to Locke in other cases in which he has deviated widely from ordinary significations and modes of expression, and has written obscurely, we rather wonder that so very little notice appears to have been taken of certian points, in this discussion, which are essential to a just appreciation of his meaning.

We had intended some further notice of Locke's statements in regard to "Ideas," in general; but we must forbear. Undoubtedly no term has been more vaguely used in psychological speculations; and this is due very much to what we may call the mystery attaching to the operations of consciousness; for every act of it presents at least two elements which are distinguishable—the conscious subject and something of which it is conscious, be the latter what it may—an external influence, an act, or state of mind. We mean that whatever be our theory of perception and of consciousness—be it in harmony

* Cours de l'Histoire de la Philosophie, T. II. Leçon 18.

† The Statute Law has varied, at different times, with respect to the punishment of offences. By the 23rd of Henry VIII., "a man becoming lunatic after an act of treason, shall be liable to be arraigned, tried, and executed." Hale says that: "If a traitor become *non compos* before conviction, he shall not be arraigned: if after conviction, he shall not be executed." (P. C. 10.) Hawkins says the same concerning those who have committed any capital offence.—(P. C. c.)

with what are called common-sense principles, or with the ancient, the Berkleian, or the modern German idealism—in thought, we are conscious, and conscious of *something* as distinct from the consciousness of it; and perhaps this general law may not have been sufficiently appreciated, explain it as we may.

We will only add, that we are glad to see an article, of a popular character, in the last number of the *Edinburgh Review*, which, while it by no means indicates insensibility to the faults of Locke, is characterized by a disposition to do him impartial justice. This, we are obliged to say, has not been done him by some of his own countrymen. With Mr. Lewes, we cannot but regret that Dr. Whewell, for instance, should have repeated the stale but most erroneous allegation that Locke "recognises no source of knowledge except the senses!" It is not surprising that a writer who held views of Locke's fundamental principle so essentially wrong, should have pronounced that he "owed his authority mainly to the intellectual circumstances of the time;" and that he "by no means possesses such metaphysical acuteness, or such philosophical largeness of view, or such a charm of writing, as to give him the high place he has held in the literature of Europe."* All who have read the *Nouveaux Essais*, will be aware in how different a tone Locke was spoken of by his most illustrious critic, Leibnitz.

* Vide quotation in Mr. Lewes's Biographical History of Philosophy, Vol. III., pp. 180, 219.

